# Optimized Conditions for Electrical Tissue Stimulation with Biphasic, Charge-Balanced Impulses

**DOI:** 10.3390/bioengineering12030234

**Published:** 2025-02-26

**Authors:** Zhengwu Sun, Payel Sen, Jules Hamers, Thomas Seidel, Andreas Dendorfer, Petra Kameritsch

**Affiliations:** 1 Walter-Brendel-Centre of Experimental Medicine, LMU Klinikum, Ludwig-Maximilians-University, 81377 München, Germany; zhengwu.sun@med.uni-muenchen.de (Z.S.); payel.sen@med.uni-muenchen.de (P.S.); jules.hamers@med.uni-muenchen.de (J.H.); andreas.dendorfer@med.uni-muenchen.de (A.D.); 2 DZHK (German Center for Cardiovascular Research), Partner Site Munich, Munich Heart Alliance (MHA), 80336 Munich, Germany; 3 Institute of Cellular and Molecular Physiology, Friedrich-Alexander University Erlangen-Nürnberg, 91054 Erlangen, Germany; thomas.seidel@fau.de

**Keywords:** field stimulation, biphasic, charge balance, tissue culture, living myocardial slice

## Abstract

The cultivation of excitable cells typically profits from continuous electrical stimulation, but electrochemical consequences are mostly harmful and must be minimized. The properties of the electrode materials and stimulation impulses are key. Here, we developed an easy method to analyze the electrochemical impact of biphasic, current-controlled impulses, applied via graphite electrodes, using phenol red as the redox indicator. We also tested the stimulation conditions for the long-term cultivation of myocardial tissue. The colorimetric assay was able to detect ±0.2% deviations in typical positive and negative pulse charges. Phenol red was best preserved (20% degradation over 24 h) by impulses of equivalent positive and negative charges (full charge balance), generated with either manual calibration, capacitive electrode coupling, or feedback regulation of electrode polarization. Feedback regulation established full charge balance at pre-pulse voltages of about 300 mV, but also provided the option to selectively compensate irreversible electrode reactions. Modifications to shape and timing did not affect the electrochemical effects of symmetric impulses. Charge-balanced stimulation maintained more than 80% of the contractility of porcine left ventricular myocardium after 10 days of culture, whereas disbalances of 2–4% provoked weakening and discoloration of the tissues. Active polarization regulation, in contrast to capacitive electrode coupling, reproduced the biological advantages of full charge balance.

## 1. Introduction

The cultivation of excitable cells has become an important research tool. To maintain the physiological function of such cells in vitro, chronic and cell-specific electrical stimulation seems to be essential [1,2]. Stimulation is usually performed by an electrical field, applied through electrodes which are not in direct contact with the cells, but are submerged in the culture medium at some distance [3]. In this indirect configuration of field stimulation, electrical gradients, e.g., at the cell membrane, are a function of local conductivity and current density, implying a direct correlation of biological effectivity and stimulation current. An effective depolarization of excitable cells typically occurs at current densities in the range of 15 mA/cm^2^ [1,4,5,6], and such currents require electrode voltages well above the redox potential of many medium constituents [7]. Consequently, electrochemical reactions at the electrode surface cannot be avoided, but must be kept to a minimum since they degrade bioactive substances, produce reactive oxygen species, or shifts in pH [7,8,9,10]. This is particularly detrimental in cell culture applications, where electrodes and solutions cannot be changed frequently, and electrochemical reaction products accumulate over time [6,7]. One way to separate electrode reactions from the cultivated cells is the transmission of currents by a defined electrolyte solution, e.g., provided by an agar bridge [11], but such designs are not compatible with the requirements of tissue culture and would not be stable over long periods. Alternatively, stimulation modalities may be chosen that take advantage of the capacitive properties of some electrode materials. The capacitance arises from the charges at the electrode surface that are reversibly bound in an ionic bilayer or as partial redox-reactions at the electrode interface [12]. As a consequence of capacitance, stimulation currents can be generated apart from irreversible electrochemical (faradaic) reactions; however, reversibility of these reactions is only ensured if the charge applied to the electrode with an electrical impulse is removed subsequently. Shortening of the electrodes after each impulse can achieve this, but typically, discharging of the electrodes is performed actively by application of an inverted current. Technically, this is implemented by biphasic impulses, which are characterized by a leading impulse, followed by an equivalent current of inverted polarity.

Recent studies employed either mono- or biphasic impulses for the long-term stimulation of cardiac cells [13,14,15,16] but information about their electrochemical impact is missing. One comprehensive investigation reported a 15% fraction as the non-recovered charge of monophasic impulses [17]. This may be important, since this charge is fully absorbed by redox reactions, possibly affecting medium components and electrode materials. Consistently, a regular exchange of electrodes has been recommended [16]. In these studies, the potential to minimize the electrochemical impact of stimulation was limited by the fact that voltage-controlled impulses were applied exclusively. With this mode of stimulation, the electrical charge of a stimulation impulse is not usually considered, and therefore, its recoverable fraction is unknown. Importantly, the biological implications of electrochemical electrode reactions are mostly disregarded.

Obviously, uncertainty exists with regard to the electrical configuration and technical implementation of field stimulation ensuring the highest biocompatibility. As stated, stimulation impulses can be generated with constant voltage or current. Voltage control is more easily implemented, but the resistance of the stimulation circuit, particularly the electrode–solution interface, may change over time. This implies alterations in the biological activity of the impulses, which directly correlates to current density. In this regard, impulses with constant current provide better control and they are a requirement for the application of a defined charge to an electrode, including its removal in an equivalent, “balanced” way. The most appropriate strength of the discharging impulse has been discussed [12]. The leading impulse will not only generate capacitive, but also irreversible currents, and it is unclear whether only reversible or total charges should be antagonized by the discharging impulse. The first option will remove the reversible charge of the electrode and therefore may prevent delayed faradaic reactions, while the second option of “fully balanced” impulses will avoid any direct current [12].

Another approach to optimize the electrochemical compatibility of field stimulation takes into account the non-linear relationship between currents and the durations of impulses with equivalent biological activities. In general, stimulation voltage and current should be kept at low but sufficient levels to guarantee stimulation of the cells [18]. However, such conditions will favor long pulse durations of the discharging impulses which might increase the total charges at the electrodes. On the other hand, short impulses will only be effective when applied with high overpotentials, and these might even produce new classes of reaction products because they exceed the redox potentials of additional culture medium ingredients. Even the frequently used symmetrical biphasic impulses may be subject to optimization, since they have been reported to exert less biological efficacy compared to monophasic impulses [18,19]. An inhibitory biological activity of the discharging pulse might be overcome by a low current and long duration of discharge, thus suggesting superior properties of asymmetric, biphasic stimulation impulses.

In the present study, the electrochemical compatibility of various configurations of field stimulation was evaluated. For the analytical part, it was assumed that faradaic reactions at the electrode surface can be quantified using phenol red as a redox-sensitive tracer. The stability of phenol red under continuous stimulation may therefore be considered as an indicator of high biological compliance with the electrical currents. Phenol red is typically used in cell culture to monitor the pH value [20]. However, in redox reactions, which can be independent of the pH value, phenol red is decolorized to transparency [21]. This characteristic can be used to measure the degradation rate of phenol red in the cell culture medium with a spectrometer which, in our case, reflects the activity of redox reactions caused by the stimulation impulses.

For the biological evaluation of various stimulation modalities, we chose their application for the long-term cultivation of myocardial tissue. It has been shown that the myocardial differentiation of stem cell-derived artificial myocardium, as well as the functional maintenance of adult myocardial tissue slices greatly profit from continuous electrical stimulation in vitro [22]. Bioreactors for the cultivation of such tissues have been designed to provide suitable biomechanical conditions, and to apply regular field stimulation [23]. In many cell culture applications, the electrical field is introduced by electrodes made of graphite, because this material is highly conductive, non-toxic, inert, and autoclavable [5]. In addition, particle-based graphite is cost-effective, and features high porosity which is associated with large specific capacitance [24]. Such electrodes were therefore used to evaluate various stimulation impulse configurations for the maintenance of living myocardial slice preparations obtained from porcine hearts. To improve the quality of long-term cultivation and stimulation of these heart slices and of bio-engineered tissues, our study pursued the following aims:

1. Development of a simple and sensitive method to quantify the electrochemical reactivity of biphasic stimulation impulses.
2. Determination of the impact of charge balance on the electrochemical properties of biphasic stimulation impulses.
3. Evaluation of capacitive electrode coupling and active impulse adaptation as the technical means to establish charge balance.
4. Evaluation of various pulse configurations with regard to the individual biological excitation efficacy and electrochemical compatibility.
5. Demonstration of the benefit of charge-balanced stimulation for the cultivation of adult pig myocardium.

## 2. Materials and Methods

### 2.1. Electrochemical Degradation of Phenol Red

A MyoDish tissue culture system designed for long term cultivation of heart slices was used in this study (InVitroSys GmbH, Gräfelfing, Germany). Each original culture chamber was filled with 2.4 mL PBS (DPBS (1×), titrated to pH 7.4, Gibco, Thermo Fisher Scientific, Waltham, MA, USA) containing 20 mg/L phenol red (Sigma-Aldrich, Merck, Darmstadt, Germany), and was incubated on the system’s integrated rocker (60/min) for 10 h or 24 h at room temperature. Electrical stimulation was performed by the MyoDish control unit, which generated constant current, biphasic impulses with arbitrary timing and PC-controlled scheduling. The initial phase of these impulses is addressed as “charging” or “stimulatory” phase, whereas the secondary phase is “discharging” the electrode capacitance. Stimulation pulses were applied at 4 Hz, using parameters well established for myocardial tissue culture (50 mA, 3 ms duration, followed by 1 ms interval and 3 ms current of inverted polarity). The stimulation frequency and duration were chosen to simulate the number of impulses applied over 3 days of slice culture at stimulation frequencies of 0.5 or 1 Hz. As such, the typical protocol for slice cultivation administered 129,600 to 259,200 impulses in between medium exchanges. To increase the sensitivity of the system, we increased the incubation time to 24 h in a set of experiments. Stimulation currents were introduced into the phenol red solution by 6 × 8 × 2 mm^3^ graphite electrodes (type CG 1290, CGC Klein, Siegen, Germany) placed at 16 mm distance After the indicated exposure to stimulation, the absorbance of the phenol red solution was determined at 430 nm with a microplate reader (SpectraMax iD3, Molecular Devices, San Jose, CA, USA). The equilibrium polarization of the electrodes was measured as the imprinted voltage present in the open-circuit condition prior to each stimulation impulse (pre-pulse voltage). This voltage was determined during the experiments with an oscilloscope (HM407, Hameg, Frankfurt, Germany). Modifications of stimulation parameters are described for the individual experiments.

### 2.2. Balance and Regulation of Stimulation Impulse Charges

**Figure 1:**
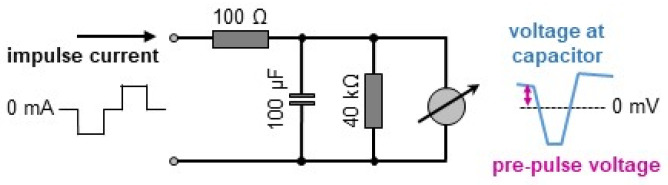
Test circuit for the manual calibration of biphasic pulses. Serial resistance and capacitance replicate electrode properties. A pre-pulse voltage of 0 V indicates equivalence of positive and negative pulse charges.

The equivalence of the negative and positive charges of each stimulation impulse was tested with an electronic model of the electrolyte interface (Figure 1). Essentially, a capacitor was charged and discharged during the respective periods of the biphasic impulses and the equivalence of both charges was assumed if no voltage was detectable during the interval between the impulses. To compensate imprecise current regulation of the impulse source, charge balance was established by manual adjustment of the duration of the positive current of each impulse. In practice, the electrode model (Figure 1) was connected to a digital voltmeter (Voltcraft VC170-1, Conrad, Munich, Germany) and to the impulse generator, which was set to standard conditions (3 ms charge and discharge, 1 ms pause, 50 mA). Pre-pulse potential was assessed as the voltage between the single negative readings provoked by the impulses (Figure 1). The duration of the positive impulse phase was stepwise shortened or prolonged (range ±200 µs) to drive the pre-pulse potential to more negative or positive values, respectively. The duration resulting in the lowest pre-pulse potential (range 0–5 mV) was accepted as the reference value of charge balance. Intentional disbalance was established by reduction or prolongation of the positive impulse phase (range −60 to +120 µs), and the relative disbalance of negative and positive charges was expressed as the deviation in its actual duration in relation to the reference value (range −2 to +4%). As an alternative way to keep positive and negative charges equivalent, we tested the suppression of any direct current by introduction of a serial capacitor into the stimulation circuit. Active control over the electrochemical consequences of biphasic stimulation was pursued by feedback regulation of the discharge pulse duration, targeting at a defined value of the pre-pulse voltage. The feedback loop was implemented with a level-shifter circuit and the internal analog–digital converter of the microcontroller (MCU).

### 2.3. Culture and Analysis of Living Heart Slices

The preparation and cultivation of living myocardial slices followed the procedures described elsewhere [5]. In short, pig hearts were obtained at the Walter-Brendel-Centre after termination of unrelated experiments. Slices of 300 µm thickness were cut from 1 cm × 1 cm transmural blocks of left ventricular myocardium using a vibratome (VT1200s, Leica AG, Wetzlar, Germany). Slices were glued to plastic holders, trimmed and mounted in MyoDish cultivation chambers with a preload tension of 1 mN. Slices were cultivated in Medium 199 (Thermo Fisher Scientific, Waltham, MA, USA), supplemented with 1% penicillin-streptomycin (Sigma-Aldrich, Merck, Darmstadt, Germany), 1% insulin-transferrin-selenium-ethanolamine (Thermo Fisher Scientific, Waltham, MA, USA), and 20 nM cortisol (Sigma-Aldrich, Merck, Darmstadt, Germany) for 10 days with constant rocking (60 rpm), and electrical stimulation (30 bpm) featuring the impulse characteristics of interest. Forces developed by the myocardial slices were continuously monitored, and the contraction force of an individual slice was derived from the amplitude of each stimulated beat. Preservation of contractility over 10 days of cultivation is given as the contraction force at the end of this period normalized to the initial value (10 h after start of cultivation, initial force). The stimulation threshold of each slice was determined by an automated protocol (starting at 50/60/70/90/120 mA for 3/2/1/0.5/0.3 ms pulse durations, respectively) which stepwise (2 mA steps in the critical region) decreased the stimulation current in 10 s intervals. The lowest current that was sufficient to maintain regular beating was considered as the stimulation threshold.

### 2.4. Statistics

One-way or two-way analysis of variance (ANOVA) was performed to test the raw data (Sigma Plot 12, Systat Software, San Jose, CA, USA). Tests were followed by comparisons versus the control (0% charge deviation) with Bonferroni’s method. The method used and the number of samples are indicated in the figure legends. Mean values and standard error of the mean are given. At an error probability of less than 5% (*p* < 0.05), the effects were considered significant.

## 3. Results

### 3.1. Full Charge Balance of Biphasic Stimulation Minimizes the Degradation of the Redox-Indicator Phenol Red

**Figure 2:**
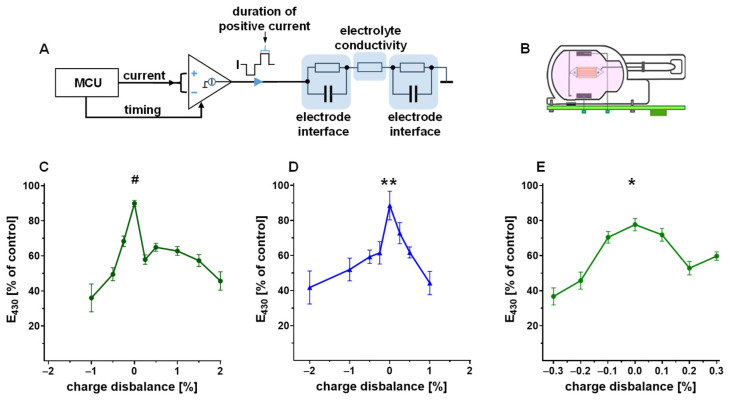
(**A**) Schematic of the impulse generator employing a microcontroller (MCU) and an adjustable current source. Highlighted in blue is the equivalent circuit of the Galvanic cell of the incubation chamber. (**B**) Design of the biomimetic chamber for tissue culture. The myocardial slice (red) is elastically mounted between the stimulation electrodes (grey rectangles). (**C**–**E**) Preservation of phenol red after stimulation for 10 h (**C**,**D**) or 24 h (**E**) with symmetric, biphasic impulses (4 Hz, 50 mA, 3 ms each phase). Impulses were generated with negative current first (**C**,**E**), or positive current first (**D**). Charge disbalance was set by shortening or prolonging the phase of positive impulse current. (**C**) # *p* < 0.01 vs. all conditions, ANOVA, Bonferroni correction, (n = 3–11); (**D**) ** *p* < 0.01 vs. ≥±1% disbalance, ANOVA, Bonferroni correction, (n = 4); (**E**) * *p* < 0.01 vs. ≥±0.2% disbalance, ANOVA, Bonferroni correction, (n = 7–10).

Biphasic impulses were applied with either equivalent (balanced) charges of the positive and negative phases, or with modifications of the duration of the positive impulse in order to generate charge disbalance (Figure 2A). In the culture chambers containing either PBS with phenol red or heart slices in the culture medium, the impulses were applied via graphite electrodes embedded in the chamber (Figure 2B). Balanced biphasic impulses (charge disbalance 0%) degraded phenol red by about 10% within 10 h regardless of whether a negative (Figure 2C) or positive (Figure 2D) current was applied first. Disbalance of positive and negative impulse charges increased the redox degradation of phenol red. Deviations of ±2% applied at 4 Hz for 10 h led to an excessive medium decolorization and decrease in phenol red absorbance by approx. 60% (Figure 2C,D). Interestingly, a positive or negative excess of impulse charges did not promote phenol red degradation in an identical manner, thereby generating a non-symmetric relationship. Impulses starting with either negative or positive currents were less active in the degradation of phenol red, when their net charge was opposite to the polarity of the leading current (Figure 2C,D). Longer (24 h) stimulation (Figure 2E) revealed that the method can detect as little as 0.2% imbalance in charges (6 µs difference in impulse durations), and confirmed a peculiar drop in phenol red stability at a 0.2–0.27% excess in the positive charge, as was also observed in the previous experiment (Figure 2C).

### 3.2. Capacitive Electrode Coupling Enforces Charge Balance and Improves the Electrochemical Compatibility of Stimulation

**Figure 3:**
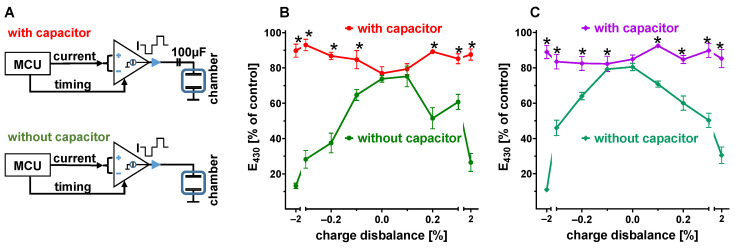
(**A**) Schematic of the pulse generator with direct or capacitive electrode coupling. (**B**,**C**) Preservation of phenol red after 24 h stimulation with biphasic impulses (4 Hz, negative current first) with or without a serial capacitor in the stimulation circuit. Impulses were either symmetric with 3 ms, 50 mA each phase (**B**), or asymmetric with a prolonged (9 ms) and attenuated (17 mA) secondary phase (**C**). * *p* < 0.01 vs. 0% direct coupling (without capacitor), two-way ANOVA, Bonferroni correction ((**B**) n = 5–13, (**C**) n = 4–16).

Biphasic impulses with a difference of up to −2% or +2% in the negative and positive charges led to a decolorization of phenol red up to 87% and 90% within 24 h, respectively (Figure 3B). Introduction of a serial capacitor into the stimulation circuit (Figure 3A) reduced phenol red degradation in all conditions tested, in a similar way as manual balancing of impulses (Figure 3B). The capacitor achieved the effect of charge balancing by accumulating the excess charge of any polarity until the impulse source could not deliver the requested charge due to its limitation in output voltage. The capacitor reduced the charge of the predominant polarity to the equivalent of the inverted impulse charge. Asymmetric stimulation with discharge prolongation (i.e., a decrease in current to one third, and an increase in duration by three-fold) had similar effects on electrochemical reactivity, as evidenced by the similarity of the minima and maxima of phenol red degradation, independent of the insertion of a capacitor (Figure 3C). A less pronounced formation of a plateau within a range of positive charge excess was the only notable difference from the symmetric impulse shape (Figure 3C).

### 3.3. Electrode Polarization Enables Active Feedback Regulation of Impulse Charges

**Figure 4:**
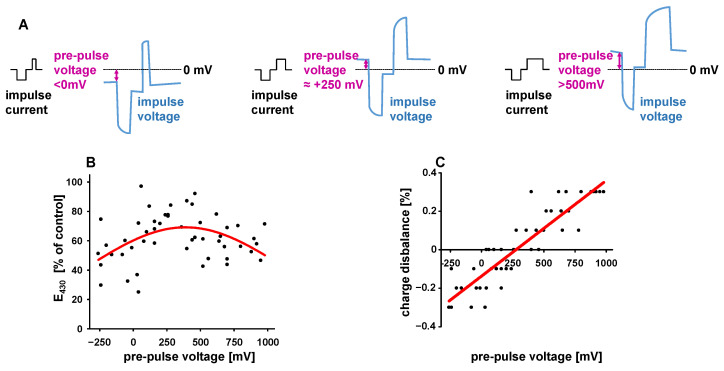
(**A**) Development of pre-pulse voltage of impulses with balanced (central graph) or disbalanced charges. (**B**) Preservation of phenol red and steady-state pre-pulse voltage after stimulation (24 h, 4 Hz, 50 mA, 3 ms, negative current first) with various states of disbalance. A non-linear fit based on a Gauss equation (red line) estimates best preservation at 390 mV. (**C**) Relationship of charge disbalance and pre-pulse voltage in the same experiment. Linear regression (red line) indicates a pre-pulse voltage of 244 mV for charge-balanced impulses (n = 5–9).

Any charge excess from repetitively applied biphasic impulses will accumulate on the electrodes until the resulting voltage will generate an equilibrium of net charge and faradaic (electrochemical) current. The voltage generated by the electrodes in an open-circuit condition can therefore be used as an indicator of charge balance. We measured the electrode voltage right before each biphasic stimulation impulse (pre-pulse voltage) under equilibrium conditions, i.e., at the end of phenol red degradation experiments. It was expected that any consistent charge excess from the stimulation impulses would shift the pre-pulse voltage to the respective polarity (Figure 4A). Indeed, there was a strict, positive correlation between both parameters, which revealed the generation of a 244 ± 55 mV baseline potential by fully charge-balanced impulses (Figure 4C). The relative recovery of phenol red confirmed this observation by demonstrating an optimum preservation in the range of pre-pulse voltages between +200 and +400 mV (Figure 4B). A pre-pulse voltage close to zero requires a charge imbalance of about −0.14% (Figure 4C), and it may be deduced from Figure 2D, that such a deviation would effectively promote phenol red degradation after 24 h.

**Figure 5:**
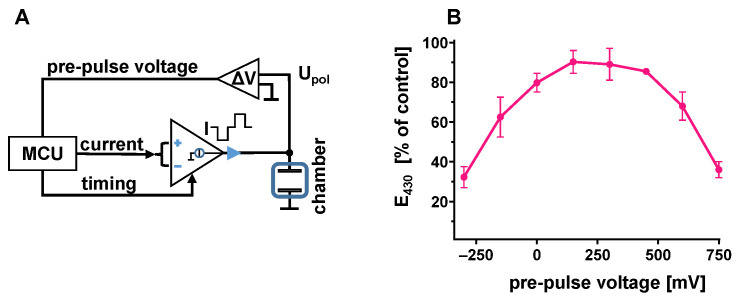
(**A**) Schematic of the active feedback regulation of pre-pulse voltage. The microcontroller (MCU) modified pulse charges in response to deviations from the pre-pulse target value. (**B**) Preservation of phenol red after 24 h stimulation (4 Hz, symmetric, 3 ms, 50 mA, negative current first) with pre-pulse voltage regulated to values of −300 to 750 mV. Best preservation was achieved with 150 mV pre-pulse voltage (n = 4).

The strict correlation between pre-pulse voltage and charge balance inspired us to utilize the easily measurable voltage as the target value for active regulation of charge balance. With the interposition of a level shifter, the pre-pulse voltage was quantified by the analog–digital converter of the microcontroller. In response to these measurements, the duration of positive current was automatically modified to generate a defined pre-pulse voltage (Figure 5A). With this regulation, phenol red degradation was studied with pre-pulse voltages actively set within a wide range (Figure 5B). Preservation of phenol red by 80–90% was observed with pre-pulse voltages between 0 and +450 mV. The optimum preservation was equivalent to the stability of phenol red achieved with manually balanced impulses (Figure 2D).

### 3.4. Stimulation Efficacies of Various Impulse Configurations in Cultivated Pig Myocardium

**Figure 6:**
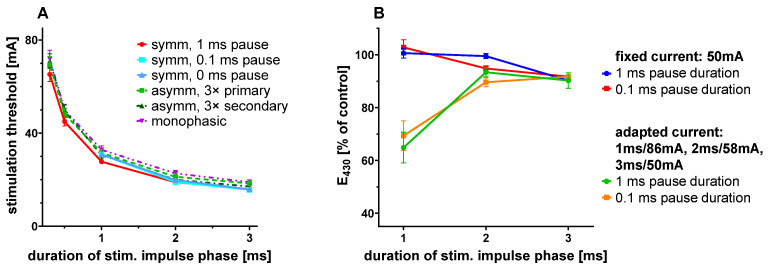
(**A**) Stimulation thresholds of impulses with various durations and shapes (symm: identical charge and discharge durations, asymm: asymmetric pulses with prolongation of duration and reduction in current by factors of 3 of either the first (3× primary) or the second (3× secondary) phase. The secondary phase was initiated after a 1 ms pause duration, if not indicated differently (n = 12–20). (**B**) Preservation of phenol red after 24 h stimulation (4 Hz, symmetric, negative current first) with pulse currents either set to 50 mA, or adapted to achieve biological efficacies equivalent to the 3 ms, 50 mA condition. Experiments were performed with either 1 ms or 0.1 ms pause intervals between negative and positive impulse phases (n = 5–11).

**Figure 7:**
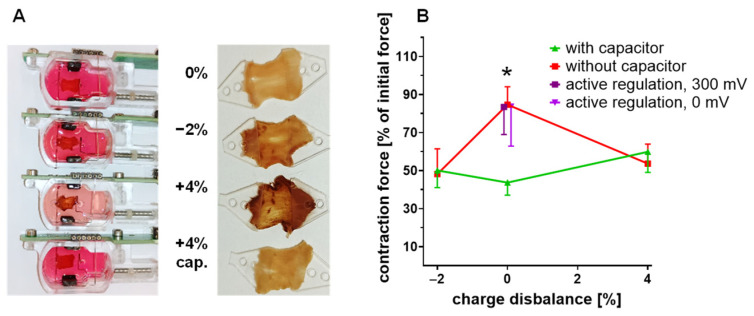
Effects of long-term stimulation on myocardial slices in tissue culture. (**A**) Typical presentation of tissue slices and cultivation medium in original biomimetic chambers after 10 days of cultivation, applying stimulation (0.5 Hz, symmetric, 3 ms, 50 mA, negative current first) with disbalanced (−2%, +4%) or balanced (0%) impulses, and direct or capacitive (cap.) electrode coupling. (**B**) Contraction forces at the end of the same experiment, as related to the initial (10 h cultivation) force of each slice. Stimulation conditions with active regulation of the pre-pulse voltages to 0 or 300 mV were included in this experiment (n = 9–19, * *p* < 0.05 vs. all conditions except active regulation, two-way ANOVA, Bonferroni’s correction).

To explore whether the biological efficacies of certain pulse configurations might be superior to those of standard symmetric, biphasic impulses, we determined their threshold intensities for the excitation of cultured myocardial slices. Thin slices of adult pig ventricular myocardium were cultured using the standard approach of the MyoDish cultivation system [5], with omission of beta-mercaptoethanol in the culture medium as the only modification. After 3–10 days of cultivation under standard stimulation (0.5 Hz, 50 mA, 3 ms biphasic balanced impulses), impulse shapes were modified, and stimulation was applied in a series of declining currents. The minimum current required to induce regular contractions of the heart slice was considered as the stimulation threshold. The stimulation threshold predicted for indefinite impulse durations is given here as the rheobase of the stimulation modality. For symmetrical impulses, the stimulation threshold (16.2 ± 0.2 mA for 3 ms, 65.2 ± 0.7 mA for 0.3 ms stimulus duration, n = 20) was lower, but very close to that of monophasic stimuli (18.9 ± 0.2 mA for 3 ms, 71.8 ± 0.9 mA for 0.3 ms stimulus duration, n = 17) (Figure 6A). Two-phase decay fitting revealed rheobase values of 14.5 mA and 16.5 mA for biphasic and monophasic impulses, respectively. Asymmetric impulses were generated with a three-fold extension of either the charge or the discharge duration and corresponding reductions in the pulse currents. Overall, their efficacies were comparable to those of symmetric impulses, indicated by rheobase values of 15.8 mA and 17.3 mA for impulses with prolonged charge or discharge durations, respectively. The overall charge of impulses was lowest using very short impulses, but, as the current required to induce stimulation was quite high in this setting, irreversible effects at the electrodes should be considered when choosing the stimulation duration for long term experiments (Figure 7B).

### 3.5. Electrochemical Compatibility of Symmetric Biphasic Impulses with Various Pulse Timings

Electrode reactions were quantified in terms of phenol red degradation over 24 h. Stimulation with balanced symmetrical impulses of 50 mA current produced more intense decolorization of phenol red with increasing pulse durations, which directly corresponded to pulse charges (Figure 6B). A reduction in the interval between negative and positive currents from 1 ms to 0.1 ms did not change this relationship significantly (Figure 6B). The reversibility of charge accumulation and faradaic reactions during the leading impulse did not seem to change within such millisecond time ranges. Short (<3 ms) impulses were also applied with increased currents to compensate for their reduced efficacy, according to the established relationship of both parameters (Figure 6A). In this condition, the high overpotential (86 mA) of the 1 ms pulse durations greatly accelerated phenol red breakdown. In summary, biphasic symmetric impulses with 2 or 3 ms pulse and 1 ms pause durations seemed to have the best ratio of effectivity and chemical reactivity of all pulse configurations investigated.

### 3.6. Electrochemical Damage in Continuously Stimulated Cultured Myocardium

Slices of porcine myocardium were constantly stimulated with balanced or disbalanced biphasic stimuli for 10 days (50 mA, 0.5 Hz, 3 ms charge and discharge with 1 ms interval). The application of severely disbalanced stimuli (+4% charge excess) led to de-colorization of the phenol red during the intervals of medium exchange (2–3 days) with no significant influence on medium pH (Figure 7A). The cultivated tissue also underwent stimulation-dependent chemical reactions, whose products accumulated over the 10 days of cultivation, and resulted in a general brownish coloration of the tissues. These changes slowly evolved during cultivation, and were clearly visible after 10 days of stimulation with either −2% or +4% charge disbalance (Figure 7A). Neither discoloration of the medium, nor brownish coloration of the tissue was observed after stimulation with charge-balanced impulses. Integration of a capacitor into the stimulation line prevented the color changes in the slices and medium even at a disbalance of 4% (Figure 7A). Under constant stimulation, the myocardial tissues contracted synchronously, and contraction force was continuously recorded by the cultivation system. Contractility was determined as the difference between diastolic and maximum systolic forces. The stable values of twitch force after 10 h of cultivation were taken as the reference for the contractility development of each slice. Contractile performance generally declined over the subsequent 10 days of cultivation, but was best preserved (>80%) when impulse charges were manually balanced, or pre-pulse voltage was adjusted to 0 or +300 mV (Figure 7B). The amplitude of contraction decreased significantly in slices treated with disbalanced stimuli (−2% or +4% deviation of net charge) and, surprisingly, charge balance by capacitive coupling did not prevent this. The capacitor in the stimulation line even deteriorated the compatibility of charge-balanced impulses.

## 4. Discussion

This study represents a practical approach to optimize the electrochemical compatibility of continuous, long-term field stimulation in a demanding in vitro environment, characterized by product accumulation and low anti-oxidative capacities. The general problem of electrochemical compliance arises from the fact that induction of electrical current in an aqueous electrolyte solution is based on either redox (faradaic) reactions or reversible ion accumulation at the electrode surface [10]. Depending on time and energies, electrode-associated redox reactions may be reversed, but the best way to prevent irreversible faradaic reactions is unclear. Here, we present an easy assay that quantifies the electrochemical compatibility of stimulation impulses using phenol red as a redox indicator, and we confirm that the application of an inverted charge in a second phase of each set of stimulation impulses is an effective way to maximize the reversible fraction of the electrode reactions. Our study indicates that the technical implementation of an impulse generator should aim for a well-controlled balance of positive and negative impulse charges, with a deviation in charges as small as 0.2% already being significant. Since charge control with this accuracy may be technically demanding, we also present a way to accomplish it by feedback regulation of electrode polarization. This technique also enables partial compensation of impulse charges, which may be beneficial when complex media are considered as electrolytes.

### 4.1. Principles of Pulsatile Field Stimulation and Charge Balance

Any current applied to an electrode will redistribute electron and ion densities at the electrode–electrolyte interface and generate an electrode potential. Static currents will raise this potential until potential-driven redox reactions will convert all electrical charges into ionic currents [12]. Biphasic stimulation with two equivalent pulses of opposing polarities provides the opportunity to minimize such irreversible electrochemical reactions by rapid discharge of the electrode capacitance and reversal of intermediate redox reactions [12]. Such discharge current can be enabled by shortening two working electrodes after termination of a monopolar impulse, but in this case, the full removal of all reversible charges will only occur at high levels of electrode polarization which will promote irreversible faradaic reactions [12]. Under such conditions, unrecoverable fractions of impulse charges as high as 15% have been reported [17]. Acceleration of electrode discharge by an inverted current subsequent to the leading impulse will reduce electrode polarization, but may provoke irreversible reactions by itself, when its inverse charge exceeds the reversible part of the leading current. Technically, this can be avoided by proper limitation of the discharging current [12].

Our study clearly indicates that full balance of positive and negative charges minimizes the electrochemical breakdown of phenol red (Figure 2B). However, the difference between the full and the reversible charge of the leading impulse is small. Charge disbalances less than 0.2% will double the degradation of phenol red compared to the balanced condition (Figure 2E). The net charge applied with this disbalance will therefore be equivalent to the faradaic loss of charges in the fully balanced condition. In this case, 99.8% of the initially applied charge will be retrieved by the discharging impulse. Consequently, discharging the electrodes to the full extent of the leading phase of the impulses (full charge balance) will leave a charge excess on each of the electrodes thereby generating an inverted polarization. This will reduce the overpotential of the leading current of the next stimulation impulse, which seems to be favorable in terms of phenol red stability even at higher degrees of charge excess, as can be concluded from the asymmetric dependency of phenol red degradation on the polarity of charge disbalance (Figure 2B,C). Equilibrium conditions are reached when each electrode has developed a baseline polarization that renders the faradaic reactions of the stimulation impulses equivalent to those within the pulse intervals. Essentially, two conditions must be considered for the irreversible part of faradaic reactions: a high redox potential during the usually short stimulatory phase of the biphasic impulse, and subsequently, a lower potential during the long interval between the impulses. Both phases may affect the degradation of various substrates differentially, according to their susceptibility to oxidation or reduction, as discussed below.

### 4.2. Electrode Reactions of Phenol Red and Electrolyte Constituents

For biological applications, the chemical nature of the electrode reactions is crucial. The effective potential at the electrode interface exceeds the redox potential of phenol red, but the respective reactions of this substrate cannot constitute a major fraction of the irreversible electrode currents. In our experiments, a total charge of 4 Hz × 0.003 s × 0.05 A × 86,400 s = 103.7 C is applied over 24 h, of which 0.2% = 0.21 C, corresponding to 1.3 × 10^18^ e^−^, are consumed by irreversible electrochemical reactions. This by far exceeds the 50.4 × 10^−6^ mol/L × 0.0024 L × 6.02 × 10^23^ parts/mol × 20% = 1.46 × 10^16^ molecules of phenol red that are degraded within this period. Under the assumption that the oxidation number of phenol red changes by 1 or 2, we would expect that only 1.1–2.2% of the irreversible faradaic reactions involve phenol red. There are several options for the electrochemical modifications underlying phenol red decolorization. Phenol red may undergo irreversible oxidation and reduction processes with complicated conformation and energy dependencies [25]. Oxidation will induce electropolymerization of phenol red, thereby forming a poly(phenol red) modification of the electrode surface [26]. Reduction may occur at a redox potential of −0.9 V by a one-electron transfer generating a radical intermediate [27].

This potential is close to the “water window” of aqueous electrolytes beyond which H_2_O hydrolysis (anodic generation of O_2_ + 4H^+^, cathodic generation of H_2_ + 2OH^−^) will occur [28]. However, graphite as an electrode material permits a greater range of electrocatalytic potentials since it demands high overpotentials of −0.47 V and +0.5 V for H_2_O hydrolysis [29]. Therefore, phenol red oxidation is unlikely to compete with H_2_O hydrolysis, but with reactions of lower redox potentials. A dominant cathodic reaction is the reduction of dissolved oxygen (O_2_ + 4H^+^ + 4e^−^ → 2H_2_O) which starts at +0.2 V electrode potential, and may include the formation of hydrogen peroxide (O_2_ + 2H^+^ + 2e^−^ → H_2_O_2_). Anodic processes may produce O_2_ from H_2_O, but at similar potentials, also reduce chlorine (2Cl^−^ → Cl_2_ + 2e^−^) with the formation of hypochlorite as a secondary product (Cl_2_ + H_2_O → HOCl^−^ + H^+^) [28]. A further anodic reaction with potential significance involves the electrode material graphite. Carbon may be oxidized by an anodic reaction (C + 2H_2_O → CO_2_ + 4H^+^ + 4e^−^) at a redox potential of +0.2 V [30]. The initial reactions of graphite, however, lead to a functionalization of the surface by generating C-OH, C=O, and CO_2_H groups, and these will require an overpotential of 0.6–1 V until degradation of graphite actually occurs [31,32]. Such reactions have been studied in simplified saline systems, but an application in cell culture will involve salt solutions with a multitude of biological supplements. Many organic substrates like carbohydrates, amino acids, and lipids are easily oxidized, so that these reactions may be predominant, particularly at low anodic potentials [28]. Due to the highly reversible nature of stimulation, the total of all irreversible reactions at balanced conditions might generate redox equivalents of 50 µM concentration per day. This may be of little significance for highly abundant medium constituents, such as glucose; however, trace amounts of vitamins, hormones and peptides may be heavily affected.

The antioxidant capacities of biological tissues and media may also explain why detrimental effects in cultured tissues occurred only under extensive charge disbalance of stimuli and after prolonged exposure. We did not further investigate the nature of brownish tissue discoloration, because it can be considered as a clearly artefactual phenomenon. The discoloration may reflect the formation of oxidized lipids, carbohydrates and proteins, and if so, it is most likely explained by the accumulation of radicals in the medium, such as peroxides and hypochlorite. In order to attenuate oxidative stress, the addition of antioxidants to the culture medium is frequently recommended. For the cultivation of heart slices, the use of 50 µM mercaptoethanol has been shown to be protective [33,34], but it has been omitted in this study to avoid electrochemical interactions. The biological significance of such interactions is still unclear, and therefore the use of mercaptoethanol should be re-evaluated, whenever novel conditions of electrical stimulation are applied.

### 4.3. Technical Implementation of Charge Balance

A variety of technical designs were developed for the specific purpose of stimulating individual muscle cells or multicellular muscle tissues [2,5,17,34]. In many cases, stimulation with voltage-controlled impulses has been successfully applied [16,17]. We opted for impulses with defined currents, because of their more direct association with the biological effects of field stimulation, and in favor of the opportunity to control impulse charges. Technically, constant current sources are readily available, and versatile control of impulse timing can be conveniently implemented with a microcontroller. Our study shows that best avoidance of electrochemical reactions requires rigorous charge balance. Unfortunately, an accuracy of pulsed currents within a range of ±0.1% are at the limit of integrated power sources and current sensors, and the specifications of custom-made or commercial stimulators in this regard are scarce. In one reported case of a neuronal stimulator, a 0.13% error of charge delivery was achieved with a specifically designed integrated circuit [35]. In the present study, the integrated amplifier LT1970 (Linear Technology, Analog Devices, Wilmington, MA, USA) was used as an adjustable current source, which is specified to ±2% accuracy of current regulation. The deviations were stable enough to be compensated manually, but they vary with the amplitude and timing of impulses, so that more general ways of charge balancing were sought.

In theory, suppression of direct current by a capacitor enforces full equivalence of positive and negative charges, with kinetic properties just depending on amplitudes and capacitance. The principle of capacitive coupling has been confirmed in our study, by the prevention of all phenol red degradation raised by disbalanced impulses (Figure 3B,C). In agreement with this observation, manually balanced impulses did not present overt alterations in shape after introduction of the capacitor. Nevertheless, the capacitor did not improve, and in the case of manually balanced impulses it even impaired, the biological compatibility with cultivated tissues (Figure 7B). The reasons for this discrepancy may be found in the non-ideal properties of real ceramic capacitors, which comprise current leakage, dielectric absorption, DC bias and serial impedance [36]. A detailed assessment of each of these peculiarities is beyond the scope of this investigation. In short, the first three of these effects should not be of major importance in our setting since they would not impact the qualities of primarily balanced impulses. A capacitor’s serial impedance includes a parasitic inductance which might generate voltage spikes in response to rapid changes of current. A quantitative estimation suggests that typical values of 1 nH and 50 mA/µs would provoke an inductive voltage of 50 µV for 1 µs duration, which is negligible in comparison to the 5 V for 3 ms duration of a typical stimulation impulse [5]. Another peculiarity related to the working principle of the capacitor is the fact that it may constitute a voltage source during the intervals between the stimulation impulses. This should not be of any consequence, provided that the stimulation circuit is fully disconnected during the intervals. Electronic switches, however, are prone to leakage currents in the range of nA, which would be driven by the charge of the capacitor throughout the pulse intervals. Such weak currents may be ineffective for the reduction in phenol red, but might affect sensitive medium constituents, e.g., ascorbic acid. This selectivity would explain the discrepancy between phenol red degradation and tissue performance which arises in the case of capacitive coupling only (Figure 5B and Figure 7B). A hypothetical baseline current would also be unidirectional and, as such, might provoke phenomena of bioelectricity. These comprise effects on cellular differentiation, proliferation, and migration, which may be provoked by very weak electrical fields [37,38]. Whether such phenomena might be relevant for the development of myocardial contractility in tissue culture needs to be determined in future studies.

Because of the ambiguities of capacitive electrode coupling, we tried to achieve charge balance by a feedback regulation using the electrode pre-pulse voltage as the regulation target. The approach relies on the strict relationship between impulse disbalance and pre-pulse potential (Figure 4C), and is able to maintain both phenol red and tissue performance as effectively as the manual adjustment of impulse charges (Figure 2C, Figure 5B and Figure 7B). Active charge balancing also provides the opportunity to compensate the irreversible loss of charges to different degrees. Appropriate adjustment, however, requires consideration of the nature and ideal magnitude of the pre-pulse voltage, which represents the added potentials of both electrodes (Figure 2A). Since the faradaic impedance is a function of polarity and potential, the latter may not be identical for the two electrodes. In the case of full charge balance, it can be stated that each impulse will place a charge excess with the polarity of the discharging current on each electrode, which will be compensated by irreversible currents during the pulse intervals. Consequently, each electrode retains an individual charge which will attenuate the electrochemical potential of the next stimulation impulse, since this will be of opposite polarity. This situation may be ideal for the prevention of reactions with high redox potentials and low diffusion limits, as presumably applies to phenol red, but it may do so at the expense of slow reactions with low redox potentials, because the sum of irreversible reactions of both electrodes will be reproduced during the intervals between the impulses. This might predominantly affect the stability of chemically susceptible substances in low abundance. In contrast, impulse adjustment to 0 mV pre-pulse potential will distribute only the difference in irreversibly lost impulse charges to both electrodes, so that their potentials during the inter-impulse phase are minimized. This situation seems favorable for the preservation of the sensitive ingredients in the culture medium. However, the differences between full and partial charge balance are small, and continuous stimulation of myocardial slices for 10 days with either condition did not reveal any superiority (Figure 7B).

### 4.4. Optimization of Impulse Shapes and Currents

Another attempt to improve the compatibility of electrical stimulation was made by modification to the impulse waveforms. The tested configurations were based on the considerations that the second phase of the biphasic impulse may attenuate the efficacy of stimulation [19], or the pause between charge and discharge may unnecessarily enhance the faradaic activity of the stimulatory phase. Neither of these hypotheses could be confirmed. The effectiveness of biological stimulation was not modified by the magnitude, current, or delay in discharge (Figure 6A). Shortening impulse durations may even enhance phenol red degradation, when the associated reduction in biological efficacy was accounted for (Figure 6B). These observations reflect the kinetics of myocardial depolarization and the exponential increase in faradaic reactions at higher electrode potentials, and indicate that bioelectrical characteristics provide no options for further improvement in the standard impulse configuration (biphasic symmetric, 2–3 ms each phase with 1 ms interval).

## 5. Conclusions

This study demonstrates that charge control is an effective measure to improve the electrochemical compatibility of biphasic electrical impulses. While its advantages have been demonstrated for the culture of adult myocardial slices, they may also apply to the chronic treatment of excitable cells (neurons, skeletal muscle cells), and to the manipulation of potential-dependent cellular functions (smooth muscle, sensory or secretory cells) in general. The observed negative impact of capacitive electrode coupling requires further investigation, since this mode is commonly used for charge balancing in medical cardiac pacemakers. For the purpose of field stimulation, the regulation of pre-pulse potential is a suitable, and probably a preferable, way to achieve charge balance. The technique is easily implemented, and avoids the technical challenge of precise current control. Assessment of electrode voltages and currents may also yield information about the redox capacities of the culture medium, thus enabling the improved control of cell metabolism and culture conditions. Technical implementations and protocols for such analyses need to be validated in future studies.

## Data Availability

The original contributions presented in this study are included in the Supplementary Materials. Further inquiries can be directed to the corresponding author.

## Supplementary Materials

The data supporting our results can be downloaded at: https://www.mdpi.com/article/10.3390/bioengineering12030234/s1, Supplementary data.
